# Costing Foodservices in Aged Care Is More Than Food Alone: The Development of the FCT

**DOI:** 10.3390/nu14142910

**Published:** 2022-07-15

**Authors:** Mikaela Wheeler, Karen Abbey, Sandra Capra

**Affiliations:** School of Human Movement and Nutrition Sciences, Faculty of Health and Behavioural Sciences, The University of Queensland, St. Lucia, QLD 4072, Australia; k.abbey1@uq.edu.au (K.A.); s.capra@uq.edu.au (S.C.)

**Keywords:** foodservice, long term care, nursing homes, malnutrition, budgets, cost analysis

## Abstract

Foodservices in residential aged-care homes (RACHs) play a vital role in providing meals and maintaining residents’ health through good nutrition. However, foodservices are often required to work within a budget, and the costs involved in foodservices are often misunderstood and underestimated. The aim of this work was to design a costing tool that included all relevant costs of a foodservice. A systems approach was used to inform the development of the Foodservice Costing Tool (FCT). Eight domains were identified, including costs that are both directly and indirectly associated with foodservices. The tool was piloted and trialled in the Australian aged-care setting and compared to currently available national estimates of costs. Through four pilots and subsequent trials, the FCT was able to capture the costs of a foodservice system in a small sample of RACHs, although the low response rate may have biased the sample toward those homes that had fewer problems with the FCT. The results highlighted the limitations of currently reported estimates, which underestimate total costs, as they fail to encompass the complexity of foodservices and to recognise that costs extend beyond the kitchen. The FCT is a useful tool and has the potential to be used by RACHs to both measure and understand their costs at a more granular level to ensure cost effectiveness and accountability. Further research is required to validate the tool and investigate the implementation of the FCT on a larger scale.

## 1. Introduction

Foodservices provide the sole source of nutrition for the majority of residents in residential aged care, and therefore they should be designed and operated in a way that meets the needs and expectations of residents. However, there are often organisational structures and budgets that impact how foodservices are provided. Foodservice managers play an important role in reconciling the needs and desires of residents with organisational budgets. Complex systems, such as those found in RACHs, can be difficult to cost; however, understanding the costs involved in these complex systems is vital to ensure efficiency and quality.

Nationally, there has been an increasing focus on costs and the level of care provided to residents in RACHs, of which the food expenditure has been highly publicised and criticised [1]. This includes the estimation of a food expenditure of $6.08 per resident per day [2]. This figure focuses on the costs of food purchases alone, which are only part of the costs involved in running a foodservice. What this figure fails to capture is the complexity of the system and the comprehensive costs that it incurs. Residents eat meals, and the cost of providing meals extends beyond simply the cost of food.

With current tools unable to accurately depict costs, there is little known about what the true costs are in running a foodservice in residential (RAC). This has repercussions for management, including difficulties in accurately measuring and understanding costs, the potential for systems to run at a higher cost than anticipated and difficulties in planning or implementing systems due to unmeasured costs [3]. In addition, without accurate data on costs, countering poor publicity in the sector is not possible. A tool that can provide a more accurate and complete estimation of costs to run a foodservice is necessary to enable services to plan, allocate resources, and operate with a higher level of efficiency. This work aimed to design a costing tool to capture the true cost of foodservices in RAC.

## 2. Materials and Methods

### 2.1. Initial Tool Development

A search of published and grey literature was conducted to identify existing tools that measured costs of hospital or RAC foodservices. It was identified that there were very few tools available to calculate foodservice costs in these settings, with most focused on hospital foodservice and on food and staff costs alone.

The Meal Unit Methodology Costing Formula created by the Institute of Hospital Catering Limited was the most comprehensive tool available, and included food, labour and kitchen supplies in its calculation [4]. As it was designed to be used in hospitals, multiple components of the tool were not applicable to the aged-care setting. The tool itself was also complex, and took a considerable amount of time to complete; however, the structure was used to inform the design of the new costing tool.

The systems approach has long been applied to the management of complex organisations, and was first applied to foodservice by Vaden [5]. When formulating the new costing tool, the systems approach was used as the basis for examining costs within a foodservice. This process utilised several foodservice management textbooks and professional knowledge to inform and examine foodservices and map costs incurred across the system [6]. The resulting tool, the Foodservice Costing Tool (FCT), measured the expenses of eight domains over a 12-month period. The domains included labour—foodservice staff, labour—non foodservice staff, maintenance, food, utilities, consumables, large equipment, and kitchen related expenses. Further explanations and inclusion for each section are outlined in Table 1. Within the domain of labour—foodservice staff, a workflow analysis was also completed with tasks grouped into the categories of kitchen administration, meal-service preparation, kitchen clean up, activity related to texture modified meals, meal delivery, meal production, staff breaks and food safety. Examples for each category are provided in Table 2.

### 2.2. Refinement of the Tool

The initial tool was tested for feasibility in calculating costs and useability by aged-care staff in three volunteer aged-care homes, located in two major capital cities in Australia between January 2019 and January 2020. Homes were both of small (<60 operational places) or medium sizes (61–100 operational places) and were operated by either not-for-profit or for-profit organisations. A convenience sample was used, with homes known to researchers approached to participate in the study. One home piloted the tool twice, with the first pilot measuring from January 2018 to January 2019 and the second pilot including January 2019–January 2020. The remaining two homes completed one pilot of the tool each for the 2018–2019 financial year. The study was conducted according to the guidelines of the Declaration of Helsinki and approved by the Ethics Committee of The University of Queensland (approval number 2019002839 date 16/12/2019). Informed consent was obtained from all participants involved in the study.

There were 4 iterations within the pilot, with feedback incorporated after each iteration. Field notes were kept by the main researcher to record issues that arose with the usage of the tool. This included questions from managers to clarify components of the tool, errors that had been made in the form and missing data. Several changes were made to the content, format, and process of using the tool over the four iterations. These are outlined in Table 3. Major changes included removing the site visit by researchers to allow all homes to use the tool regardless of location; alterations to the format to reduce the time associated with completing the tool; the addition of examples to reduce errors made within the tool; the addition of a domain (large equipment purchases) to reduce the over-inflation of the consumables domain and the inclusion of supplements in the food domain, rather than a separate domain. Data analysis was completed using Microsoft Excel 2016. Once the tool was completed, homes were provided with a report of their foodservice costs.

### 2.3. Validation of the Tool in Practice

After piloting the tool, researchers aimed to validate the FCT using a number of homes of varying size, location and ownership type. Snowballing was used to recruit homes. The study was advertised through multiple channels including through personal networks, through advertisement in newsletters of three national aged-care or foodservice organisations and through social media. No restrictions were placed on the type or number of homes that could participate. While all homes were eligible to participate and were going to be provided with an individualised cost report, a subgroup of homes was going to be selected to be included in the final analysis. This subgroup was to include an appropriate mix of homes that considered their location, bed number and ownership type to ensure the final group would be representative of aged-care homes within Australia. Due to the low participation of providers, there was an inadequate number of homes to complete analysis in this way.

Three RAC providers (*n* = 26 homes) provided consent to participate in the first recruitment phase; however, due to the rise of COVID-19 in early 2020, a number of participating homes withdrew or paused their enrolment in the study. One provider withdrew 10 homes, and another requested a pause on data collection in its 15 homes. Only one home continued with data collection from this phase. Recruitment was paused during this time due to the large focus on pandemic management for RACHs and anticipated low levels of expression of interest in participating.

Recruitment recommenced in January 2021, through the previously mentioned channels. Nine providers (*n* = 16 homes) expressed interest in participating in the study and seven provided written consent (*n* = 14 homes). Fourteen homes were emailed the FCT forms. Following subsequent reminders, two providers (*n=* 3 homes) did not return their forms. This was considered as withdrawing from the study. Five providers (*n=* 11 homes) partially completed the forms, and only one home completed the entire foodservice costing tool and received a report of their foodservice from phase two of recruitment. Across both phases of recruitment, only two homes completed the FCT. Demographics for homes in the pilot and validation studies are listed in Table 4. Data were collated and analysed using Microsoft Excel 2016.

## 3. Results

### 3.1. Pilot Results

Three homes completed the pilot of the FCT. Total foodservice expenditure ranged from AUD $27.91 to $49.75 per resident per day (prpd) (Table 5). The range was smaller, however, for food cost, ranging from AUD $9.37 to $10.71 prpd (Table 5). The largest costs within a foodservice were, in order, labour (foodservice staff), food, and labour (non-foodservice staff). The costs to run foodservices in Home A were significantly lower than other homes, which may be partially explained by incomplete data collection due to difficulty in follow up. There was also a large increase in foodservice costs in Home B between the two data points due to a large increase in time spent on mealtime assistance, with the number of staff in dining rooms doubled and mealtimes increased from 30 min to 45 min.

#### 3.1.1. Foodservice Activities Completed by Non-Foodservice Staff

The allocation of activities to non-foodservice staff varied between homes, with most hours spent on mealtime assistance. On average, mealtime assistance was provided for 45 min at the three main meals. Smaller mealtimes, including morning tea, afternoon tea and supper, took on average 15 min. Activities such as setting and packing up the dining room, taking residents’ orders, meal delivery and meal ordering were regularly undertaken by non-foodservice staff in each home, with an associated cost ranging from AUD $2.38 to $8.65 prpd (Table 5). This constituted the third largest expense for each home’s foodservice.

#### 3.1.2. Workflow Analysis

An analysis of foodservice staff workflows was completed to understand how staff time was spent in foodservices. Results showed that for each home, the most time was spent on activities relating to kitchen clean up. The second highest was meal production, followed by meal-service preparation and kitchen administration. Activity related to texture-modified meals consumed the least amount of time. Collecting workflow data was difficult for each home, with workflows either non-existent or not reported in enough detail to allow for granular analysis. In order to obtain this information, significant time was spent by researchers creating personalised forms for each home.

### 3.2. Validation Results

Two homes completed the FCT (homes D and E in Table 4) and received a report of the costs for their foodservice. Total foodservice expenditure ranged between AUD $40.02 and $54.85prpd, with the three largest expenses being labour (foodservice), food and labour (non-foodservice staff). Food expenditure ranged from AUD $9.98 to $11.36 per resident per day (Table 5).

#### 3.2.1. Foodservice Activities Completed by Non-Foodservice Staff

For both homes D and E, time was spent by non-foodservice staff across all foodservices activities, with the exception of meal ordering and dining room setup for home D. Between sixteen and eighteen minutes were spent on these activities per resident per day, with an associated cost of AUD $6.85–$8.54. Similarly to the results in the pilot phase, expenditure for staff not directly employed in the foodservice was the third largest expense for homes.

#### 3.2.2. Workflow Analysis

Similarly, to the homes in the pilot phase, the majority of foodservice staff time was spent on kitchen cleanup and meal production. However, meal delivery was the third largest task, followed by meal-service preparation. Foodservice hours were spent differently in Home E, however, with meal-service preparation taking the most time, followed by meal production, meal delivery and kitchen cleanup. Both homes spent the least amount of time on activities related to texture modification. Like homes in the pilot phase, obtaining workflow data from homes was difficult. There were no existing workflows in either case, and the researchers had to help create them.

## 4. Challenges

This study encountered a number of challenges, including low recruitment rates, high dropout or non-completion rates and issues with specific parts of the tool.

### 4.1. Low Recruitment

The first challenge encountered was the low recruitment numbers. Despite significant advertisement in prominent industry publications and the extensive reach of the research team, recruitment was still low. Noting the complex system that aged care exists within and its permeability to the external environment, field notes were kept to record external events and their potential impact on the system at the time of the study. These were explored to understand possible reasons for low recruitment and non-completion rates. Possible reasons included the increased workload (actual or perceived) due to the pandemic, multiple snap or prolonged lockdowns across multiple states, a sector under stress due to a national Royal Commission into Aged Care Quality and Safety, increased scrutiny from the media and changes to funding, reporting requirements and accreditation (increased unannounced visits). All of these external events placed significant strain on the aged-care system, and provide potential reasons as to why RACHs may not have engaged in research. It is also noted that Stewart Brown Chartered Accountants completes annual financial benchmarking for 1200 RACHs (44% of Australian RACHs), which includes an estimation of food and foodservice staff costs. With this information already provided to a large percentage of RACHs, it is possible that there was not a perceived need for the FCT.

### 4.2. Missing Data

While five providers failed to complete the FCT, all provided some data. To further understand whether there were areas of the tool that were more difficult to complete than others, an analysis of the submitted data was completed. When mapped across areas of the tool, it became clear that costs for utilities, supplements and non-foodservice staff were most commonly not included. To further explore these costs, an influence diagram was created to understand what determines the total cost of foodservices within RAC. From this analysis, it became evident that while these categories contribute to the cost of the foodservice, they traditionally sit outside the foodservice itself, potentially increasing the difficulty of obtaining this information. An example is staff allocation for nursing and care staff, who may assist in taking residents’ orders, or assist at mealtimes or supplement expenditure. These costs traditionally sit within nursing; however, they are interrelated with foodservice costs. Difficulty in obtaining information from other parts of the system was also evident in communication with homes. Most cited a lack of access to data or difficulty in obtaining data from other people outside the foodservice.

## 5. Discussion

The FCT was developed to calculate the total cost of foodservices in RACH due to the limited scope of current tools. Across all trials (*n* = 6), the average spend on foodservices was AUD $42.65 per resident per day (range $27.91–$54.85) Food expenditure ranged from AUD $9.37 to $11.36 per resident per day, which is consistent with the costs published by Stewart Brown for the same year [7]. There were a number of challenges encountered in the design and validation of the tool, including the format, content and process of using the tool as well as low recruitment numbers and challenges in obtaining data. The tool was used to capture the cost of foodservice in a small sample of aged-care homes. Through pilot and validation phases, the FCT was able to differentiate where costs might be increased, decreased or redirected. This type of information can deliver baseline data to provide management with confidence in decision making.

Understanding the complexity of foodservices and how this translates into costs is very important in ensuring system efficiency; however, current tools and reported figures underestimate the true cost of running foodservices. Hugo, C [2], reported food costs of AUD $6.08 per resident per day in 2016. This increased to $8.00 per resident per day when including supplements, meal replacements and consumable items, such as crockery, cutlery and paper goods. Since publication, however, Stewart Brown Chartered Accountants, who collated the data used in this study, have stated that ‘reports that the daily food content is in the range of $6.50 per resident are incorrect’ [8]. There is a misrepresentation of costs due to the fact that one third of the homes included used a contract catering model, and therefore the costs of food and consumables were included in the contract catering price and not the food cost, resulting in an underestimation of costs [7].

Since then, costs have been reported differently by Stewart Brown in order to improve transparency and accuracy. Costs are reported as total catering costs, which are made up of consumables, staff and contract catering costs, which are differentiated as either in-house catering or contract catering. In the 2021 financial year report, average total catering costs were reported as AUD $33.10 prpd, with very little difference between in-house ($33.09) and contract catering ($33.14). The average amount spent on food, supplements and cooking ingredients was AUD $12.92 prpd [8]. This was an increase from the 2020 financial year report, with total catering costs of AUD $32.00 prpd, and an average spend on food, supplements and cooking ingredients of $12.50 [9].

Costs reported by the FCT are higher than currently reported costs, which is to be expected due to the inclusion of a larger number of foodservice costs in its calculation. However, if these costs, including non-foodservice staff, maintenance, utilities, large equipment and kitchen-related expenses, are removed for comparison, it can be seen that the total foodservice costs for the majority of homes (*n* = 4) are similar to the Stewart Brown figures (AUD $30.66–$39.08). This provides a degree of confidence in the tool to correctly account for costs, and highlights the additional costs that are included in the FCT.

The Resource Utilisation and Classification Study (RUCS), completed by the University of Wollongong, proposed a new assessment and funding model for residential aged care in Australia. While it did not provide specific costs, it did identify cost drivers (the clinical and need characteristics of aged-care residents that influence the cost of care) within the system. Costs were classified as structural or fixed costs. Fixed costs are those that are related to the characteristics of the facility rather than individual care needs, such as general supervision at mealtimes. Variable costs are the cost of care that is required to address the individual care needs of residents. This study found that facilities that are remote or small, or that specialise in Indigenous care, had higher fixed care costs [10]. When comparing this to the results of the FCT, this was the case for home E, which was classified as both small and remote, and had the highest total foodservice cost per resident per day of AUD $54.85.

A difference between the FCT and the currently reported figures is the inclusion of costs associated with mealtime activities that are completed by non-foodservice staff, such as supervision or providing assistance at mealtimes. The RUCS study identified that mealtimes contribute to both the fixed and individual costs. The Resource Utilisation Groups-Activities of daily living (RUG-ADL) is designed to profile loss of function using, eating, transfers, toileting and mobility. It measures the resources required to carry out respective functional tasks. An analysis of independent variables showed that function, mobility, and activities of daily living (including eating) were the largest cost drivers for residents [11]. It is therefore important that these costs are considered within the foodservice.

Since the commencement of this work, there have been changes in funding related to food and nutrition as a result of the Royal Commission into Aged Care Quality and Safety. The Royal Commission highlighted a number of areas in which the aged-care system required improvement, one of which was food and nutrition. Recommendation 112 proposed immediate changes to the Basic Daily Fee by providing an additional $10 per resident per day [12]. This supplement was introduced on the 1 July 2021with the aim of supporting residential aged-care providers to deliver better care and services to residents with a focus on food and nutrition. In order to continue receiving this supplement, RACHs must report quarterly on their food and nutrition expenditure and the quality of daily living services. Figures that are mandatory to report include expenditure on food, ingredients, preprepared and bought-in main meals. Expenditure on oral nutrition supplements, oral health living expenses, hours for cooks, chefs and other food management or foodservice staff, and expenditure on allied-health support for residents to improve their nutritional wellbeing, do not currently have to be provided, however they will become mandatory for the quarterly financial report commencing in October 2022 [13]. This reporting does not include other necessary costs of running a foodservice, including equipment, consumables, utilities, general kitchen expenses and the cost of staff to support residents at mealtimes. The figures will also not be made publicly available, and therefore it will not be possible to use these data to benchmark against other services.

### Limitations

While this study adds to the literature around costs of foodservices in residential aged care, there are limitations to its findings. The major limitation of this work is the low recruitment and the subsequent small sample used to pilot and test the FCT. Due to external events, such as the Royal Commission and COVID-19, recruitment was difficult. The study also saw high dropout and non-completion rates. Whether this was due to external events or the use of the tool itself is unknown. Due to the low response rate, the sample might not be representative. In particular, homes that had problems with the FCT might have been less willing to complete and return it. Further research is required to investigate the use of the tool in RAC and its applicability to practice.

Due to the prolonged impact of COVID-19, data collection spanned multiple years. This may impact the ability to compare different costs between each of the homes, due to potential changes in the prices of goods. The complexity of foodservices and the need to rely on facility staff to report figures does affect the precision and accuracy of results. While the sample of homes used was small and not representative of Australian aged-care homes, the FCT measured costs in the aged-care setting and identified components of foodservices contributing to overall costs. Further studies are needed to validate the tool and recruit a larger, more representative sample of homes to determine the average spend on foodservices in Australia.

## 6. Conclusions

While the development and testing of the tool faced many barriers, the final FCT was able to capture the costs of RACH foodservice for a small and possibly unrepresentative sample of homes. It is the first tool to identify the complexity of foodservices and recognise that costs are located within different parts of the residential aged-care system. By ensuring homes are accurately reporting and attributing their costs to all parts of the foodservice, the FCT enables services to demonstrate cost-effectiveness and to potentially justify and plan future system changes in order to meet the needs and expectations of consumers now and into the future.

## Figures and Tables

**Table 1:** Inclusions for each domain.

Domain	Inclusions
Labour (foodservice staff)	All staff directly employed within the foodservice and involved in the following activities: meal production, kitchen cleaning and maintenance, setting and clearing dining rooms, serving resident meals, assisting residents with eating, meal delivery and meal ordering.
Labour (non-foodservice staff)	All staff who are not employed directly in the foodservice and who are involved in the following activities: setting and clearing dining rooms, serving resident meals, assisting residents with eating, meal delivery and meal ordering.
Maintenance	Maintenance of equipment, such as servicing and repairs, waste removal and laundry associated with food services.
Food	Including fruit, vegetables, dairy, meat, poultry, bakery, dry and frozen goods, prepared foods and supplements, including pre-made supplements, powders, juices and desserts.
Utilities	Any utilities used by the kitchen. 6. Gregoire, M.B. [6] estimates that foodservices in schools and hospitals use 5%–10% of the building’s total energy usage. A conservative approach was taken, and 10% of utility costs was adopted for the tool.
Consumables	Includes items such as cleaning materials, disposables, such as straws, napkins, cups etc., and small kitchen equipment including utensils, crockery, cutlery, pots, pans and glasses.
Large equipment	Includes items such as large kitchen equipment (dishwashers, ovens etc.) and meal delivery equipment.
Kitchen related expenses	Examples include office supplies, uniforms, staff training and menu management or accounting software.

**Table 2:** Description of workflow activities.

Activity Category	Activity Examples
Food Safety	Checking of storage temperatures, dishwasher temperatures, pre-service food-temperature checks, recording of temperatures, food safety plan.
Kitchen Administration	Ordering food, stocktakes and unpacking deliveries.
Meal-Service Preparation	Loading trolleys, dishing up meals, preparing crockery, utensils, unpacking trolleys and setting up areas required for meal service. Setting up and packing down dining rooms.
Meal Production	Any activity that contributes to the cooking of a meal, including garnishing.
Texture Modified	Any activity that solely contributes to preparation of texture modified meals, i.e., blending, moulding, labelling, reheating.
Staff Breaks	Including major break and minor break.
Meal Delivery	Delivering meals to dining rooms and residents’ rooms and serving meals to residents.
Kitchen Cleanup	Washing dishes, cleaning kitchen and cleaning equipment.
Meal Ordering	Collecting residents’ menus or verbal orders.

**Table 3:** Variations made over each iteration.

Variations	Iteration Number			
	1	2	3	4
In-person audits of mealtimes completed	✓	✓		
Home provided with the list of required documents at the site visit	✓			
List of required documents sent to home prior to site visit		✓		
Removal of site visit			✓	✓
Addition of pre-determined answers with tick boxes, examples, extra space for explanations			✓	✓
Simplified format and removal of questions			✓	
Section added for large equipment purchases			✓	
Transfer of Mealtime Activities section to excel document				✓
Addition of completed excel example				✓
Wording of ‘position description’ altered to workflow			✓	
Example of workflows provided				✓

**Table 4:** Residential aged-care demographics.

RAC	Size Classification ^1^	ABS Remoteness Classification ^2^	Foodservice System	Management of Services ^3^
A	Small	Major City, Queensland	Cook Fresh	For profit
B	Medium	Major City, Queensland	Cook Fresh and Cook chill	For profit
C	Medium	Major City, South Australia	Cook Fresh	Not for profit
D	Medium	Outer Regional Australia, New South Wales	Cook Fresh	Not for profit
E	Small	Outer Regional Australia, New South Wales	Cook Fresh	Not for profit

^1^ Small = 60 or fewer operational places, Medium = 61–100 operational places and Large = 101 or more operational places. ^2^ Australian Bureau of Statics Remoteness Classification. ^3^ Management of services in Australia are operated by not-for-profit (religious, charitable and community organisations), government or private organisations.

**Table 5:** Total expenditure across each FCT domain figure.

Expenditure Type	Home A	Home B * 1	Home B * 2	Home C	Home D	Home E
Labour (foodservice staff)	$8.87	$26.18	$26.91	$22.61	$19.89	$31.39
Labour (non-foodservice staff)	$8.55	$2.38	$8.65	$7.96	$6.85	$8.54
Maintenance	$0.15	$1.24	$0.85	$0.23	$0.31	X^2^
Food (including supplements)	$9.38	$9.87	$10.57	$9.86	$9.98	$11.36
Utilities	$0.73	$0.49	$0.53	$0.62	$0.39	$0.59
Consumables	$0.19	$0.79	$1.60	$0.58	$0.79	$1.42
Large Equipment	X^1^	X^1^	$0.18	$0.00	$1.52	$1.29
Kitchen Related Expenses	$0.06	$0.42	$0.46	$0.14	$0.29	$0.26
**Food Cost Resident/day**	$9.37	$9.87	$10.71	$9.86	$9.98	$11.36
**Total Foodservice Cost Resident/day**	$27.91	$41.37	$49.75	$41.99	$40.02	$54.85

* Costs represented as per resident per day in AUD. Across the data collection period, the consumer price index (CPI) rose by between 0.7% and 1.8% per quarter. X^1^ Included in maintenance X^2^ Included in consumables. Retain bold—these were highlighted because they are the main and most important figures in the table and ones that are referred to throughout the manuscript.

## Data Availability

Not applicable.
